# Characteristics and comorbidities of headache in patients over 50 years of age: a cross-sectional study

**DOI:** 10.1186/s12877-022-03027-1

**Published:** 2022-04-10

**Authors:** Mansoureh Togha, Mohammad Javad Karimitafti, Zeinab Ghorbani, Fatemeh Farham, Fereshteh Naderi-Behdani, Somayeh Nasergivehchi, Zahra Vahabi, Shadi Ariyanfar, Elham Jafari

**Affiliations:** 1 Headache Department, Iranian Center of Neurological Research, Neuroscience Institute, Tehran University of Medical Sciences, Tehran, Iran; 2 Department of General Surgery, Shahid Sadoughi University of Medical Sciences and Health Services, Yazd, Iran; 3 Department of Cardiology, Cardiovascular Diseases Research Center, Heshmat Hospital, School of Medicine, Guilan University of Medical Sciences, Rasht, Iran; 4 Department of Clinical Nutrition, School of Medicine, Guilan University of Medical Sciences, Rasht, Iran; 5 Department of Neurology, School of Medicine, Alborz University of Medical Sciences, Karaj, Iran; 6 Department of Neurology, Baharloo Hospital, Tehran University of Medical Sciences, Tehran, Iran; 7 Department of Geriatric Medicine, Ziaeian Hospital, Tehran University of Medical Sciences, Tehran, Iran; 8 Department of Clinical Nutrition and Dietetics, Faculty of Nutrition and Food Technology, Shahid Beheshti University of Medical Sciences, Tehran, Iran

**Keywords:** Headache, Middle-age and older, Migraine, Tension-type headache, Depression, Sleep apnea, Medication-overuse headache

## Abstract

**Background:**

Although headache is a common complaint in younger individuals, it is one of the most common complaints among persons over the age of 50 and is a significant cause of morbidity. As there are differences in the causes and types of headache, the diagnosis and management of headache in older adults differ from that in younger individuals.

**Methods:**

In this cross-sectional study, 570 patients ≥ 50 years were recruited at a university affiliated tertiary headache center between 2016 and 2019. Demographic data, headache characteristics, and comorbid medical conditions were recorded. The presence of depression was explored using the Beck Depression Inventory. The patients were evaluated using the STOP-BANG scale to determine the risk of obstructive sleep apnea.

**Results:**

The mean age of the patients was 57.7 years. Seventy-three percent of the patients had primary headache disorders, with the most prevalent types being migraine, followed by tension-type headache. Secondary headaches were primarily the result of overuse of medication, cervical spine disease, and hypertension. Patients with medication-overuse headache were significantly more likely to suffer from hypothyroidism and gastrointestinal problems such as bleeding/ulcers. Irritable bowel syndrome was also more common in patients with medication-overuse headaches and migraines. The risk for obstructive sleep apnea was intermediate in 45.2% of the patients with hypertension-induced headache, but was lower in the majority of others. There was a high tendency for moderate-to-severe depression in the participants; however, the Beck Depression Inventory scores were significantly higher in medication-overuse headache patients.

**Conclusion:**

Proper treatment of headache in middle-aged and older adults requires the recognition of secondary causes, comorbid diseases, and drug induced or medication overuse headaches. Special attention should be paid to depression and obstructive sleep apnea in such patients suffering from headache disorders.

**Supplementary Information:**

The online version contains supplementary material available at 10.1186/s12877-022-03027-1.

## Key Points

- The most common type of primary headaches in individuals over the age of 50 were episodic migraines and TTH.
- Depression scores were significantly higher in patients with MOH.
- A relationship was found between OSA and HTN-induced headache.

Why does this paper matter?

As the information regarding headache disorders in people over the age of 50 is scarce, this research might improve understanding about headache characteristics and comorbidities in this age group.

## Background

Headache is the tenth most common symptom in older women and the fourteenth most common one in older men [1]. The overall prevalence of headache decreases to 12- 50% with age; however, careful consideration of secondary causes of headaches, comorbidities, altered drug pharmacokinetics and polypharmacy is required in this age group [2–4].

In older adults, the majority of the headaches are primary headache disorders (66%); however, there is a higher risk of secondary causes (up to 15%) compared to younger adults [2, 3, 5]. Tension-type headache (TTH) is the most common type in older adults, with an annual prevalence of 16% to 44% in patients over the age of 50 years [6–8]. The clinical symptoms of TTH do not change with age, but the percentage of secondary headaches misdiagnosed as TTH can increase in older adults [5–11]. Migraine is the second most common primary headache disorder in this population, with a prevalence of 5% to 10% [7, 11, 12]. A new-onset migraine headache is unusual after the age of 60 years and should motivate prompt evaluation for a secondary cause [3, 5–8, 12]. Episodic migraines can transform into chronic migraines in older patients, especially in the presence of other comorbidities [5, 9, 12].

The most common secondary causes of headache in this age group are medication-overuse and trauma to the head and/or neck. Approximately 15% of all headaches in patients over 65 are associated with medication use [13]. Headache is a common side-effect of many medications and use of analgesics more than 10–15 days/month for more than three months could cause medication-overuse headache (MOH) [5]. Cervicogenic headache is another secondary headache that is believed to be more prevalent in older adults because of their higher prevalence of degenerative cervical disk disease [2]. The incidence of sleep apnea is considered to peak in middle-aged adults; however, its prevalence continues to increase with age and it is an important cause of headaches that could be overlooked [3].

Patients over the age of 50 who complain of headache more often have comorbidities [14, 15]. These comorbid health problems can complicate the diagnosis of a headache, limit treatment options, and increase the risk of progression to chronic headaches. Many comorbidities have been reported in association with migraine, including cardiovascular disorders, psychiatric disorders, neurological diseases, sleep disorders, and inflammatory and chronic pain conditions [16]. Headache patients are at risk of depression and a depressed patient will suffer from more frequent headache episodes. Anxiety and depression are comorbid with migraine, MOH, and TTH; this should be considered when selecting the appropriate treatment options for both conditions [12].

Information about headache disorders in middle-aged and older people is scarce; most of the available studies have been carried out in younger patients. This cross-sectional study was conducted to evaluate the characteristics and comorbidities of headache in patients of ≥ 50 years of age at a tertiary headache center from 2016 to 2019. Special attention was paid to comorbid psychiatric disorders, including depression, and sleep apnea through the use of relevant questionnaires.

## Methods

### Study population

In this cross-sectional study, 3265 patients presented to a headache referral clinic between April 2016 and March 2019 complaining of headache. Of these, 610 patients were aged 50 years and older. All headache sufferers in this age group were included in the current study, except for patients with impaired cognitive function, language problems, or any other serious medical or psychiatric condition as based on the physician’s evaluation. The final total of 570 patients who were consecutively recruited were able to provide a reliable history that was often confirmed by a relative.

All of the participants reported at least two headache episodes in the last three months that were not related to an acute oral, dental, or paranasal sinus infection or eye problems. The patients underwent clinical evaluation by expert neurologists/headache specialists and the types of headache were diagnosed according to the criteria of the International Classification of Headache Disorders-3 (ICHD3) [17]. The participants who reported more than one form of headache received a diagnosis for each particular condition. If a secondary headache was suspected, brain and cervical imaging, including the vasculature and the blood and/or cerebrospinal fluid (obtained via lumbar puncture) were examined based on the headache specialist’s recommendation.

### Data collection

#### Demographic, anthropometric, and headache-related data

The patient demographic data (age, sex, occupation, education level, marital status), anthropometric data (weight, height, body mass index (BMI)) and smoking and opium use were recorded. Information was collected about headache-related features such as headache location, duration (< 3 min, 3–15 min, 15–180 min, > 4 h), frequency (1, 2–4, or > 5 days/week), associated symptoms, and other headache characteristics and comorbidities as well as medication history for headache treatment.

#### Medical history

To obtain the required data for the medical history, drug history, and presence of comorbidities, the patients were asked to complete a questionnaire. If more information was required, their medical records were reviewed. Otherwise, the common signs and symptoms of the disorders were self-reported by the participants. The comorbidities and concurrent diseases included were cardiovascular disorders, hypertension (HTN), as well as thyroid, kidney, gastrointestinal, cervical spinal and temporomandibular disorders. The drug history included tricyclic antidepressants (TCAs), selective serotonin reuptake inhibitors (SSRIs), analgesics, opioids, anxiolytics, antiepileptic drugs (AEDs), corticosteroids, calcium channel blockers (CCBs), beta blockers, angiotensin-converting-enzyme inhibitors (ACEIs), diuretics, statins and other types of cardiovascular medications, anti-diabetic drugs, and antihistamines.

#### Beck Depression Inventory

The depression status was explored using the validated translation of the Beck Depression Inventory (BDI), a multiple-choice self-reporting 21-item questionnaire [18, 19]. Each of the BDI-scale items has a four-point answer (from 0 to 3) indicating depression intensity that provides a total score of 0 to 63 as the sum of all answers. The patients were categorized into six groups according to the defined level of depression as normal or minimal depression (a score of 1–10), mild mood disturbance (a score of 11–16), borderline clinical depression (a score of 17–20), moderate depression (a score of 21–30), severe depression (a score of 31–40), and extreme depression (a score of > 40) [18, 19].

#### STOP-BANG sleep apnea questionnaire

All patients were evaluated using the snoring, tiredness, observed apnea, [high] blood pressure, BMI, age, neck circumference, and [male] gender (STOP-BANG) scale to determine the risk of obstructive sleep apnea (OSA). This scale contains eight yes/no questions. A “yes” response has a score of 1 and a “no” response has a score of zero. The total score ranges from 0 to 8 and is used to classify patients as low risk (total score of 0–2), intermediate-risk (total score of 3–4), or high risk (total score of 5–8).

### Statistical methods

All statistical analyses were conducted in SPSS 24.0 (IBM; USA). Descriptive data such as frequency, percentage, mean, and standard deviation (SD), were arranged into tables and graphs when appropriate. The Pearson’s chi-square test, one-way analysis of variance (ANOVA), and the Bonferroni post-test were applied to examine statistically significant between-group differences in categorical data and continuous variables, respectively. A *p*-value < 0.05 was considered as statistically significant.

## Results

A total of 570 patients, including 511 women (89.6%) and 59 men (10.4%) aged 50 + years were included in the study. The mean age of the patients was 57.7 years. Of these, 398 patients were between 50–60 years and 172 subjects were above the age of 60 years.

Seventy-three percent of the patients had primary headache disorders. The majority of patients suffered from episodic and chronic migraine (45.2%), followed by TTH (21%) as the most prevalent types of primary headaches. Secondary headaches were found in about 27% of patients. The most prevalent secondary headaches were MOH, cervicogenic, and HTN-induced. The diagnoses by the headache specialist of 570 subjects showed that 198 (34.7%) had episodic migraine, 120 (21.1%) had TTH, 63 (11.1%) had MOH, 61 (10.7%) had chronic migraine, 47 (8.2%) had cervicogenic headache, 42 (7.3%) had HTN-induced headache, 24 (4.2%) had cluster headache, and 15 (2.6%) had hemicrania continua. HTN-induced headache was diagnosed if the headache occurred in close temporal relation to hypertension and completely resolved after appropriate treatment of the HTN. Four patients suffered from trigeminal neuralgia and one patient was diagnosed with pituitary macroadenoma. These patients were not included in the final analysis.

The demographic data of the study population was compared according to headache type. It was observed that patients in the episodic migraine group were significantly younger than the other patients. In contrast, subjects suffering from HTN-induced headache, hemicrania continua, and cluster headaches were slightly older than the other patients (*p* < 0.001).

The patients who were diagnosed with TTH, cervicogenic headache, and HTN-induced headache were more likely to have a higher BMI. In comparison, those with episodic migraine had a significantly lower BMI compared to TTH sufferers (*p* = 0.019). The proportion of males was significantly higher in patients diagnosed with cluster or hemicrania continua (*n* = 13; 33.3%), followed by TTH (*n* = 18; 15%) and MOH (*n* = 9; 14.3%) (*p* < 0.001). Between-group comparison showed no significant differences in marital status, smoking, and opium use between patients diagnosed with the various types of primary or secondary headache.

Most patients stated that their headaches had begun before the age of 50 (*n* = 417; 73%); however, 47.6% of the patients who had HTN-induced headache, 41% of patients with hemicrania continua or cluster headache, and 40.8% of those with TTH experienced their first episode after 50 years of age. Most patients with episodic migraine, TTH, HTN-induced, and cervicogenic headache experienced headaches on fewer than four days per week, while those with chronic migraine and MOH experienced headaches on five or more days per week (Table 1).

**Table 1:** Overview of demographic characteristics of the study population according to headache type

**Variable**		**Episodic migraine (*n* = 198)**		**Tension type headache (*n* = 120)**		**Hemicranias continua or cluster headache (*n* = 39)**		**chronic migraine (*n* = 61)**		**HTN-induced headache (*n* = 42)**		**Cervicogenic headache (*n* = 47)**		**Medication overuse headache (*n* = 63)**		***P*-value **^**#**^
**Age, year**		55.53 * # ¥ ‡	5.21	59.08 *	8.31	60.90 # €	12.87	58.88	8.16	60.34¥	7.97	59.38 ‡	10.47	55.89 €	5.36	** < 0.001**
**Body mass index, kg/m2**		25.94 *	3.98	27.51 *	4.19	26.24	3.81	26.71	4.57	27.98	4.29	27.61	3.71	27.03	4.56	**0.019**
**Male Sex**		8	4.0%	18	15.0%	13	33.3%	3	4.9%	5	11.9%	3	6.4%	9	14.3%	** < 0.001**
**Education**	** < 10 years**	50	25.3%	51	42.5%	18	46.2%	28	45.9%	13	31.0%	21	44.7%	10	15.9%	**0.001**
	**10–12 years**	56	28.3%	35	29.2%	10	25.6%	13	21.3%	11	26.2%	11	23.4%	21	33.3%	
	**12–14 years**	19	9.6%	9	7.5%	4	10.3%	6	9.8%	8	19.0%	9	19.1%	4	6.3%	
	**14–16 years**	47	23.7%	16	13.3%	4	10.3%	9	14.8%	7	16.7%	5	10.6%	18	28.6%	
	** > 16 years**	26	13.1%	9	7.5%	3	7.7%	5	8.2%	3	7.1%	1	2.1%	10	15.9%	
**Job**	**Employee**	43	21.7%	15	12.5%	9	23.1%	9	14.8%	10	23.8%	6	12.8%	15	23.8%	**0.002**
	**Self-employed**	15	7.6%	26	21.7%	7	17.9%	7	11.5%	10	23.8%	5	10.6%	4	6.3%	
	**retired**	40	20.2%	30	25.0%	11	28.2%	12	19.7%	12	28.6%	7	14.9%	17	27.0%	
	**Un-employed/ housewife**	100	50.5%	49	40.8%	12	30.8%	33	54.1%	10	23.8%	29	61.7%	27	42.9%	
**Marital status**	**Single**	5	2.5%	5	4.2%	1	2.6%	1	1.6%	0	0.0%	4	8.5%	1	1.6%	0.237
	**Married**	175	88.4%	98	81.7%	36	92.3%	52	85.2%	39	92.9%	37	78.7%	61	96.8%	
	**Divorced**	12	6.1%	11	9.2%	0	0.0%	5	8.2%	3	7.1%	4	8.5%	1	1.6%	
	**Other**	6	3.0%	6	5.0%	2	5.1%	3	4.9%	0	0.0%	2	4.3%	0	0.0%	
**Smoking**		15	7.6%	19	15.8%	7	17.9%	7	11.5%	4	9.5%	4	8.5%	8	12.7%	0.491
**Opium use**		2	1.0%	5	4.2%	3	7.7%	2	3.3%	0	0.0%	2	4.3%	3	4.8%	0.204

Data are presented as mean and standard deviation or number and percentage as appropriate

^#^Results derived from chi-square test or one-way ANOVA

^* # ¥ ‡^ Matching symbols indicate significant between-group differences

Few specific disease states were found to be associated with these headaches. As expected, HTN was significantly more prevalent in patients with an HTN-induced headache. MOH patients were significantly more likely to suffer from hypothyroidism, gastrointestinal bleeding, and/or peptic/duodenal ulcer (*p* < 0.05). About 30.2% of those diagnosed with MOH and 25.8% of those diagnosed with migraine had IBS. Other disorders were not significantly different between study groups (Table 2).

**Table 2:** Comorbidities of the study population according to headache type

**Variable**		**Episodic migraine** **(*n* = 198)**		**Tension type headache** **(*n* = 120)**		**Hemicranias continua or cluster headache** **(*n* = 39)**		**chronic migraine** **(*n* = 61)**		**HTN-induced headache** **(*n* = 42)**		**Cervicogenic headache** **(*n* = 47)**		**Medication overuse headache (*n* = 63)**		***P*-value **^**#**^
**Cardiovascular disorders**		26	13.1%	25	20.8%	9	23.1%	13	21.3%	11	26.2%	13	27.7%	11	17.5%	0.166
**Hypertension**		41	20.7%	36	30.0%	14	35.9%	20	32.8%	31	73.8%	10	21.3%	20	31.7%	** < 0.001**
**Thyroid disorders**	**Hypothyroidism**	50	25.3%	34	28.3%	7	17.9%	15	24.6%	8	19.0%	10	21.3%	28	44.4%	**0.012**
	**Hyperthyroidism**	38	19.2%	17	14.2%	5	12.8%	21	34.4%	7	16.7%	4	8.5%	3	4.8%	
	**Thyroid nodules**	4	2.0%	1	0.8%	0	0.0%	0	0.0%	1	2.4%	1	2.1%	2	3.2%	
**Renal disorders**	**Renal failure**	3	1.5%	1	0.8%	0	0.0%	1	1.6%	3	7.1%	0	0.0%	0	0.0%	0.084
	**Kidney stones**	20	10.1%	16	13.3%	4	10.3%	6	9.8%	5	11.9%	4	8.5%	14	22.2%	
**Gastrointestinal disorders**	**diarrhea**	34	17.2%	14	11.7%	10	25.6%	6	9.8%	2	4.8%	6	13.0%	9	14.3%	0.112
	**History of gastrointestinal bleeding and/or** **Peptic/duodenal ulcers**	23	11.6%	17	14.2%	2	5.1%	9	14.8%	5	11.9%	2	4.3%	16	25.4%	**0.024**
	**Irritable bowel syndrome (IBS)**	57	28.8%	28	23.3%	5	12.8%	10	16.4%	7	16.7%	8	17.0%	19	30.2%	0.088
**Cervical spine disease**	**disc disorders**	31	15.7%	9	7.5%	1	2.6%	7	11.5%	3	7.1%	2	4.3%	11	17.5%	0.103
	**Degenerative disorders**	56	28.3%	40	33.3%	11	28.2%	20	32.8%	17	40.5%	16	34.0%	24	38.1%	
**Clenched teeth / Bruxism**		34	17.2%	21	17.5%	12	30.8%	12	19.7%	13	31.0%	6	12.8%	12	19.0%	0.066

Data are presented as number and percentage

^#^Results derived from chi-square test

**Fig. 1:**
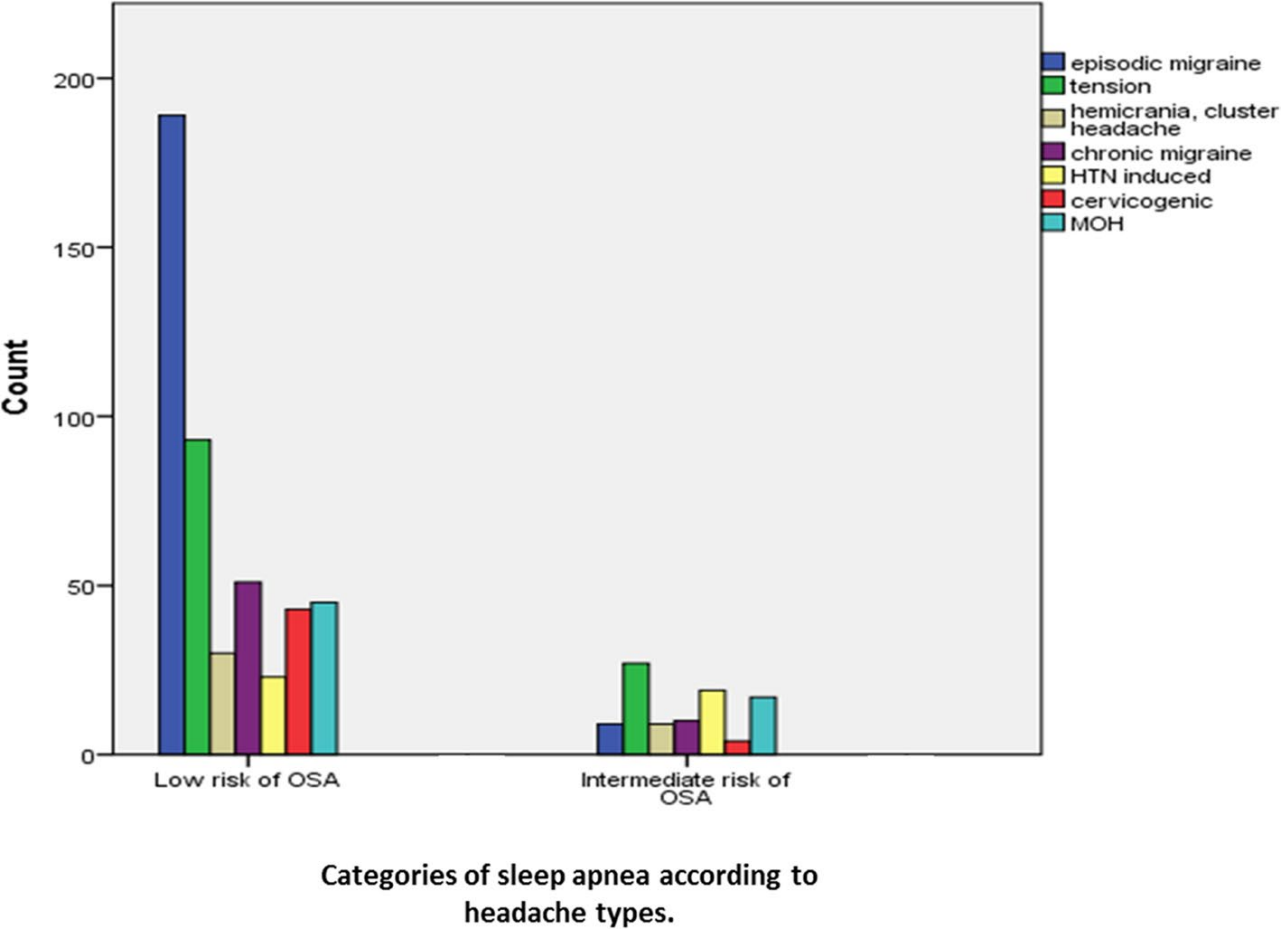
Distribution of patients according to categories of sleep apnea total score based on STOP-BANG questionnaire

Figure 1 shows the distribution of patients according to the total OSA-score categories based on the STOP-BANG questionnaire. It was found that the risk of OSA was intermediate in about 45.2% [19] of patients who were diagnosed with HTN-induced headache, whereas the risk was lower in the majority of other patients.

The mean (SD) score of the BDI was 28.29 (10.55) for patients with episodic migraine, 28.95 (10.87) for TTH, 25.37 (11.70) for chronic migraine, 28.81 (9.78) for hemicranias continua or cluster headache, 28.88 (11.37) for HTN-induced headache, 25.49 (12.90) for cervicogenic headache, and 33.39 (11.11) for MOH. One-way ANOVA showed significant between-group differences in the mean BDI scores. MOH patients had significantly higher BDI scores than those with episodic migraine, chronic migraine, and cervicogenic headache (*p* = 0.003). The chi-square test showed that the majority of MOH patients (> 90%) had moderate, severe, or extreme depression. Overall, there was a high tendency for moderate to severe depression in patients aged 50 years and over who were diagnosed with headache in the current study (Table 3).

**Table 3:** Categories of depression based on Beck Depression Inventory (BDI) score according to headache type

	**Episodic migraine** **(*n* = 198)**		**Tension type headache** **(*n* = 120)**		**Hemicranias continua or cluster headache** **(*n* = 39)**		**chronic migraine** **(*n* = 61)**		**HTN-induced headache** **(*n* = 42)**		**Cervicogenic headache** **(*n* = 47)**		**Medication overuse headache (*n* = 63)**		***P*-value **^**#**^
**Normal**	7	3.6%	5	4.6%	1	2.7%	5	8.3%	2	4.8%	7	15.6%	2	3.3%	**0.009**
**Mild mood disturbance**	14	7.2%	7	6.5%	3	8.1%	6	10.0%	3	7.1%	3	6.7%	0	0.0%	
**Borderline clinical depression**	19	9.7%	11	10.2%	2	5.4%	11	18.3%	3	7.1%	0	0.0%	2	3.3%	
**Moderate depression**	78	40.0%	37	34.3%	13	35.1%	18	30.0%	17	40.5%	13	28.9%	19	31.1%	
**Severe depression**	59	30.3%	34	31.5%	14	37.8%	13	21.7%	11	26.2%	20	44.4%	23	37.7%	
**Extreme depression**	18	9.2%	14	13.0%	4	10.8%	7	11.7%	6	14.3%	2	4.4%	15	24.6%	

^#^Chi-square test

### Subgroup analysis

The majority of headaches in patients between 50–60 years were primary headache disorders, with episodic migraine (*n* = 169, 42.5%), being the most common diagnosis. Besides, among those over 60 years of age tension type headache (*n* = 47, 27.3%) was diagnosed as the most prevalent headache type (Supplementary materials, Table 1). A subgroup analysis was conducted in order to explore the differences in demographic characteristics, comorbidities distribution, depression status based on BDI score and sleep apnea total score based on STOP-BANG among the patients between 50–60 years (*n* = 398) and those older than 60 years (*n* = 172) which is presented as Supplementary materials, Tables 2 (A, B) to 6 (A, B). Concerning the comparison of depression and sleep apnea according to headache types among these age group, no differences were detected in BDI scores in neither among 50–60 years group and nor among those who were older than 60 years (Supplementary material Table 5, A and B). Moreover, the majority of included headache patients in both groups showed a low risk of OSA, except for the subjects who aged older than 60 years and had HTN-induced headache. These subjects demonstrated a higher prevalence of intermediate risk of OSA than other headache patients (*P*-value < 0.001) (Supplementary material Table 6, A and B),

## Discussion

Headache disorders are among the most prevalent of disabling conditions worldwide. In Iran, headache is a common disorder with significant morbidity. A cross-sectional study of 23,457 individuals in Tehran found a prevalence of 6.9% for chronic headache [20]. In a systematic review study, the overall prevalence of migraine in an Iranian population was 14%, which was similar to or even higher than many other parts of the world [21]. The prevalence of headache in older adults ranges from 5 to 50% globally [1].

The present study described headache characteristics, subtypes, and comorbidities among patients aged 50 years and above who presented to referral headache clinics. Women constituted 89.6% of the cases. This higher prevalence of headache disorders in women has been demonstrated in previous studies at 72% to 78% [13, 14].

Primary headaches were diagnosed in 73% of patients, which is consistent with the results of previous studies of 66% to 84% [3, 5, 13, 14]. The most frequent diagnosis in our population was episodic migraine followed by TTH. In subgroup analysis, the most common diagnosis in patients younger than age 60 was episodic migraine, while TTH was the most common diagnosis in patients over age 60, followed by migraine. These results are similar to a study in Malaysia that divided patients into older (55 years and above) and younger (less than 55 years of age) subjects. In their study, TTH was the most common subtype among the older age group and migraine without aura was more common in young adults [22].

Other studies have reported similar results for migraine and TTH in patients over 65 years [14]; however, TTH was the most common diagnosis (28% to 45%) in most previous studies [2, 11, 13]. Although TTH is more common in the older adult population, its prevalence might be lower in patients presenting to headache clinics because it is less severe and disabling. The higher prevalence of episodic migraine in the current study could relate to the referral nature of the study setting (a headache center) and the study population as being 50 + years of age. This is likely to include the peri-menopausal years in some participants, which has been associated with migraine exacerbation. Sixty-one patients in our series (10.6%) met the diagnostic criteria for chronic migraine, while other studies have reported chronic migraine in 15% to 26% of older patients [13, 14].

Most of the primary headache disorders appear before the age of 50 and a few of them occur almost exclusively in people over the age of 50. It has been reported that primary headaches initiate over the age of 65 years in 4.8% of cases[23]. In the present study, headaches started after 50 years of age in 27% of participants, including 47% of those with HTN-induced headache, 41% of those with cluster or hemicrania continua, and 40.8% of those with TTH.

Several studies have found a higher percentage of secondary headaches in older individuals (10% to 30%) [3, 13, 14, 24]. About 27% of the patients in the present study were diagnosed with secondary headaches. The most common types were MOH, cervicogenic headache, and HTN-induced headache. Lisotto et al. [14] reported that, in their study, the most frequent secondary headaches were cervical spine disorders (26.2%), trigeminal neuralgia (26.2%), and cervicogenic headache (23.7%). Ruiz et al. [13] reported secondary headaches in 16% of their patients aged over 65 and were most frequently attributed to substance use, followed by cranial trauma. The overuse of analgesics is a common practice among the older people (30% to 45%) because of the increased prevalence of chronic pain conditions [25]. Medication-overuse headache was diagnosed in 11% of our patients, and nearly all of them had concurrent chronic migraine which led to the medication overuse. In another study of patients aged over 65, 37.8% of those reporting chronic daily headache had medication overuse, with a higher proportion suffering from chronic migraine rather than chronic TTH [6].

The overall prevalence of cervicogenic headache is estimated to be 1% to 4% [26]; however, the true incidence is unknown in patients over 65 years because it is often mistaken for TTH [24]. Forty-seven patients (8.2%) in our study met the diagnostic criteria for cervicogenic headache. However, cervical spine disorders, including discopathy or degenerative changes, were found in 44% of these patients, which might have affected their headaches. In a study by Lisotto et al. [14], 23.7% of secondary headaches were attributed to cervicogenic headache.

Middle-aged and older individuals more often have comorbid disorders [14, 15]. We found a number of specific disease states that were associated with headache. The results showed a significantly higher prevalence of hypothyroidism in patients with MOH. In a large population-based study evaluating comorbidity of migraine with somatic disease, both hyperthyroidism and hypothyroidism were comorbid with migraine [27]. Another study in Egypt also showed a significantly higher proportion of subclinical and overt hypothyroidism in patients with migraine and TTH compared to the control groups [28].

MOH patients had higher rates of gastrointestinal bleeding and/or peptic/duodenal ulcers (25.4%), which could be explained by the overuse of analgesic medication. About 30.2% of those diagnosed with MOH and 25.8% with migraine also had IBS. This association between migraine and functional gastrointestinal disorders such as IBS in the present study is consistent with the results of many other studies [29]. In a prospective cohort of migraine patients in Korea, 40.4% fulfilled the diagnostic criteria for IBS [30].

Most studies have found a positive association between migraine and arterial HTN [31]. Approximately 30% of the patients in the present study had HTN. As expected, HTN was significantly more prevalent in patients with HTN-induced headache in the current study.

Psychological factors, including depression and anxiety, have long been suspected to be linked to headache [32–34]. In the present study, most subjects (80%) were found to have moderate or severe depression. A cohort study of older adults in Iran also found an independent association between headache and psychological factors, particularly in women [35]. In their study, headache was reported in 42% and depression in 42.4% of subjects. The high rates of depression in the present study could be explained to some extent by the referral nature of the study setting, the significant number of patients with chronic migraine and MOH, and the delay in diagnosis of some headache subtypes, especially cervicogenic headache, which could have led to disability and depression.

Depression and anxiety disorders have been associated with both migraine and non-migraine headaches, especially TTH, in several studies [33, 36]. The results of the present study showed that MOH patients had significantly higher BDI scores compared to patients with episodic migraine, chronic migraine, and cervicogenic headache. Similarly, the findings of the Eurolight project [33] indicated stronger relationships between anxiety in particular, as well as depression, and MOH.

Current data support the comorbidity of obesity with headache in general and migraine specifically [37]. The present study found higher BMI scores in patients who were diagnosed with TTH, cervicogenic headache, and HTN-induced headache. On the other hand, those with episodic migraine had a significantly lower BMI compared to TTH sufferers. A study in China reported that chronic migraine patients were more likely to have a higher BMI than episodic migraine patients, while episodic TTH patients were more likely to be overweight/obese compared to chronic TTH patients [38].

Another influencing factor on development or aggravation of headaches is sleep apnea, although it is not studied quietly and most data relates to cluster headache. The result of studies on the role of sleep apnea on migraine headache are not conclusive. The present study was in line with other studies indicating no clear relationship between migraine and risk of OSA. However, subjects who aged older than 60 years and had HTN-induced headache were at intermediate risk for obstructive sleep apnea according to the STOP-BANG questionnaire, whereas the risk of obstructive sleep apnea was lower in the majority of other patients. A large body of evidence supports this bidirectional relationship between OSA and chronic HTN. The prevalence of HTN is 50% to 60% in patients with OSA, and 30% to 40% of hypertensive patients also have OSA [39]. Hence, OSA and HTN require prompt diagnosis and treatment to reduce the risk of morbidity.

The present study had some limitations because it was cross-sectional with no long-term follow-up and some secondary headaches in older people may initially be misdiagnosed as TTH. As our study cases were from the tertiary headache clinics, they may not be representative of the general population. However, the advantages of this study include: (1) evaluation of a significant number of patients; (2) investigation of the different comorbidities affecting headaches, especially OSA and depression, using questionnaires and face-to-face interviews and; (3) diagnosis of headache types by an expert neurologist according to the ICHD3 criteria.

## Conclusion

As headaches are more common in a young population, most studies have focused on this age group, but headaches in a large number of middle-aged and older individuals could indicate significant morbidity. In the present study, primary headaches were found in 73% of patients, with the most common types being episodic migraine and TTH. Secondary headaches were found in 27% of patients, with the most common causes being medication overuse, cervical spine disease, and HTN. Regarding comorbidities, functional gastrointestinal disorders were more common in patients with episodic migraine and MOH.

The majority of our patients were rated with moderate or severe depression and the depression scores were significantly higher in patients with MOH. Depression should be specifically considered in the treatment of patients, because concomitant treatment of depression is likely to improve headache control and quality of life. There was no clear relationship between migraine and OSA; however, subjects who aged older than 60 years and had HTN-induced headache were at intermediate risk for obstructive sleep apnea, which supports a bidirectional relationship between OSA and chronic HTN. Consequently, for proper treatment of headache in older adults, it is necessary to rule out secondary causes and focus on the presence of concomitant diseases and medications.

## Supplementary Information

**Additional file 1: Supplementary material Table 1. **Distribution of various headache types according to age categories. **Supplementary material Table 2. **Mean age and body mass index (BMI) of the study population according to headache type. **Supplementary material Table 3. **Overview of demographic characteristics of the study population according to headache type. **Supplementary material Table 4. **Comorbidities of the study population according to headache type. **Supplementary material Table 5. **Categories of depression based on Beck Depression Inventory (BDI) score according to headache type. **Supplementary material Table 6. **Distribution of patients according to categories of sleep apnea total score based on STOP-BANG questionnaire.

## Data Availability

The datasets used and/or analysed during the current study are available from the corresponding author on reasonable request.

## References

[1] Ozge A (2013). Chronic Daily Headache in the Elderly. Curr Pain Headache Rep.

[2] Starling AJ (2018). Diagnosis and Management of Headache in Older Adults. Mayo Clin Proc.

[3] Bravo TP. Headaches of the Elderly. Current Neurology and Neuroscience Reports. 2015;15(6). 10.1007/s11910-015-0552-2 25893722

[4] Ward TN (2002). Headache Disorders in the Elderly. Curr Treat Options Neurol.

[5] Walker RA, Wadman MC (2007). Headache in the Elderly. Clin Geriatr Med.

[6] Prencipe M, Casini AR, Ferretti C, Santini M, Pezzella F, Scaldaferri N (2001). Prevalence of headache in an elderly population: attack frequency, disability, and use of medication. J Neurol Neurosurg Psychiatry.

[7] Schwaiger J, Kiechl S, Seppi K, Sawires M, Stockner H, Erlacher T (2009). Prevalence of primary headaches and cranial neuralgias in men and women aged 55–94 years (Bruneck Study). Cephalalgia.

[8] Camarda R, Monastero R (2003). Prevalence of primary headaches in Italian elderly: preliminary data from the Zabut Aging Project. Neurol Sci.

[9] Straube A, Andreou A. Primary headaches during lifespan. The Journal of Headache and Pain. 2019;20(1). 10.1186/s10194-019-0985-0 PMC6734460 30961531

[10] Crystal SC, Grosberg BM (2009). Tension-type headache in the elderly. Curr Pain Headache Rep.

[11] Berk T, Ashina S, Martin V, Newman L, Vij B (2018). Diagnosis and Treatment of Primary Headache Disorders in Older Adults. J Am Geriatr Soc.

[12] Haan J, Hollander J, Ferrari MD (2007). Migraine in The Elderly: A Review. Cephalalgia.

[13] Ruiz M, Pedraza MI, de la Cruz C, Barón J, Muñoz I, Rodríguez C (2014). Headache in the elderly: a series of 262 patients. Neurología (English Edition).

[14] Lisotto C, Mainardi F, Maggioni F, Dainese F, Zanchin G (2004). Headache in the elderly: a clinicalstudy. J Headache Pain.

[15] van Oosterhout WPJ, Cheung C, Haan J (2016). Primary headache syndromes in the elderly: epidemiology, diagnosis and treatment. J Clin Transl Res.

[16] Buse DC, Reed ML, Fanning KM, Bostic R, Dodick DW, Schwedt TJ (2020). Comorbid and co-occurring conditions in migraine and associated risk of increasing headache pain intensity and headache frequency: results of the migraine in America symptoms and treatment (MAST) study. J Headache Pain.

[17] Headache Classification Committee of the International Headache Society (IHS) The International Classification of Headache Disorders, 3rd edition. Cephalalgia : an international journal of headache. 2018;38(1):1–211. 10.1177/0333102417738202 29368949

[18] Beck AT, Ward CH, Mendelson M, Mock J, Erbaugh J (1961). An inventory for measuring depression. Arch Gen Psychiatry.

[19] Beck AT, Steer RA, Carbin MG (1988). Psychometric properties of the Beck Depression Inventory: Twenty-five years of evaluation. Clin Psychol Rev.

[20] Mohammadzadeh F, Faghihzadeh S, AsadiLari M, VaezMahdavi MR, Arab Kheradmand J, Noorbala AA (2015). A Fairly Comprehensive Survey of Chronic Pain in Iranian Population: Prevalence, Risk Factors, and Impact on Daily Life. Health Scope..

[21] Farhadi Z, Alidoost S, Behzadifar M, Mohammadibakhsh R, Khodadadi N, Sepehrian R (2016). The Prevalence of Migraine in Iran: A Systematic Review and Meta-Analysis. Iran Red Crescent Med J.

[22] Tai M-LS, Jivanadham JS, Tan CT, Sharma VK (2012). Primary headache in the elderly in South-East Asia. J Headache Pain.

[23] Kunkel RS (2006). Headaches in older patients: special problems and concerns. Cleve Clin J Med.

[24] Sharma TL (2018). Common primary and secondary causes of headache in the elderly. Headache: J Head Face Pain.

[25] Tonini MC, Bussone G (2010). Headache in the elderly: primary forms. Neurol Sci.

[26] Robblee J, Singh RH (2020). Headache in the Older Population: Causes, Diagnoses, and Treatments. Curr Pain Headache Rep.

[27] Le H, Tfelt-Hansen P, Russell MB, Skytthe A, Kyvik KO, Olesen J (2010). Co-morbidity of migraine with somatic disease in a large population-based study. Cephalalgia.

[28] Abou Elmaaty AA, Flifel ME, Belal T, Zarad CA (2020). Migraine and tension headache comorbidity with hypothyroidism in Egypt. Egypt J Neurol, Psychiatr Neurosurg.

[29] Lau C-I, Lin C-C, Chen W-H, Wang H-C, Kao C-H (2014). Association between migraine and irritable bowel syndrome: a population-based retrospective cohort study. Eur J Neurol.

[30] Park JW, Cho Y-S, Lee SY, Kim E-S, Cho H, Shin HE (2013). Concomitant functional gastrointestinal symptoms influence psychological status in Korean migraine patients. Gut and liver.

[31] Finocchi C, Sassos D (2017). Headache and arterial hypertension. Neurol Sci.

[32] Wei C-B, Jia J-P, Wang F, Zhou A-H, Zuo X-M, Chu C-B (2016). Overlap between Headache, Depression, and Anxiety in General Neurological Clinics: A Cross-sectional Study. Chin Med J (Engl).

[33] Lampl C, Thomas H, Tassorelli C, Katsarava Z, Laínez JM, Lantéri-Minet M, et al. Headache, depression and anxiety: associations in the Eurolight project. The journal of headache and pain. 2016;17:59-. 10.1186/s10194-016-0649-2 PMC4887397 27245683

[34] Uthaikhup S, Sterling M, Jull G (2009). Psychological, cognitive and quality of life features in the elderly with chronic headache. Gerontology.

[35] Ahmadi Ahangar A, Hossini S-R, Kheirkhah F, Bijani A, Moghaddas Z (2016). Associated factors of headache in an unstudied cohort of elderly subjects. Caspian J Intern Med.

[36] Cohen CI, Henry KA (2011). The prevalence of headache and associated psychosocial factors in an urban biracial sample of older adults. Int J Psychiatry Med.

[37] Chai NC, Scher AI, Moghekar A, Bond DS, Peterlin BL (2014). Obesity and headache: part I–a systematic review of the epidemiology of obesity and headache. Headache.

[38] Huang Q, Yu H, Zhang N, Guo B, Feng C, Wang S (2019). Body Mass Index and Primary Headache: A Hospital-Based Study in China. Biomed Res Int.

[39] Chahal CAA, Somers VK (2015). Secondary hypertension: obstructive sleep apnea. J Am Soc Hypertens.

