# Supplementary material for: Characteristics and comorbidities of headache in patients over 50 years of age: a cross-sectional study
Source: BMC Geriatr. 2022 Apr 10;22:313. doi: 10.1186/s12877-022-03027-1 (PMC8994908; doi:10.1186/s12877-022-03027-1)
Supplement: Supplementary file 1 — Additional file 1: Supplementary material Table 1. Distribution of various headache types according to age categories. Supplementary material Table 2. Mean age and body mass index (BMI) of the study population according to headache type. Supplementary material Table 3. Overview of demographic characteristics of the study population according to headache type. Supplementary material Table 4. Comorbidities of the study population according to headache type. Supplementary material Table 5. Categories of depression based on Beck Depression Inventory (BDI) score according to headache type. Supplementary material Table 6. Distribution of patients according to categories of sleep apnea total score based on STOP-BANG questionnaire. [file 12877_2022_3027_MOESM1_ESM.docx]

**Supplementary materials**

**Supplementary material Table 1. Distribution of various headache types according to age categories.**

|  | **Number of patients** | **Percentage** |
| --- | --- | --- |
| **Patients between 50-60 years (n=398)** | | |
| **Episodic migraine** | 169 | 42.5% |
| **Tension type headache** | 73 | 18.3% |
| **Hemicranias continua or cluster headache** | 17 | 4.3% |
| **MOH and Chronic migraine** | 91 | 22.9% |
| **HTN-induced headache** | 23 | 5.8% |
| **Cervicogenic headache** | 25 | 6.3% |
| **Patients older than 60 years (n=172)** | | |
| **Episodic migraine** | 29 | 16.9% |
| **Tension type headache** | 47 | 27.3% |
| **Hemicranias continua or cluster headache** | 22 | 12.8% |
| **MOH and Chronic migraine** | 33 | 19.2% |
| **HTN-induced headache** | 19 | 11.0% |
| **Cervicogenic headache** | 22 | 12.8% |

**Supplementary material Table 2- Mean age and body mass index (BMI) of the study population according to headache type.**

1. **Among patients between 50-60 years (n=398)**

|  | **Categories of headache type** | | | | | | | | | | | | |  | |
| --- | --- | --- | --- | --- | --- | --- | --- | --- | --- | --- | --- | --- | --- | --- | --- |
|  | **Episodic migraine** | | **Tension type headache** | | **Hemicranias continua or cluster headache** | | **MOH and Chronic migraine** | | **HTN-induced headache** | | **Cervicogenic headache** | | | **P-value from ANOVA** | |
|  | Mean | Standard Deviation | Mean | Standard Deviation | Mean | Standard Deviation | Mean | Standard Deviation | Mean | Standard Deviation | Mean | Standard Deviation |  | |  |
| **Age, year** | 53.87 | 3.47 | 53.75 | 5.43 | 51.24 | 12.02 | 53.79 | 3.24 | 54.14 | 2.61 | 52.24 | 7.88 | 0.217 | |  |
| **Body mass index, kg/m2** | 25.78 ^a, b, c^ | 4.05 | 27.62 ^a^ | 3.96 | 26.60 | 3.83 | 27.08 ^b^ | 4.43 | 26.80 | 4.56 | 27.69 ^c^ | 3.35 | 0.032 | |  |

1. **Among** **patients older than 60 years (n=172)**

|  | **Categories of headache type** | | | | | | | | | | | | |  | |
| --- | --- | --- | --- | --- | --- | --- | --- | --- | --- | --- | --- | --- | --- | --- | --- |
|  | **Episodic migraine** | | **Tension type headache** | | **Episodic migraine** | | **Tension type headache** | | **Episodic migraine** | | **Tension type headache** | | | **P-value from ANOVA** | |
|  | Mean | Standard Deviation | Mean | Standard Deviation | Mean | Standard Deviation | Mean | Standard Deviation | Mean | Standard Deviation | Mean | Standard Deviation |  | |  |
| **Age, year** | 65.14 | 2.52 | 67.13 | 4.56 | 68.36 | 7.45 | 66.91 | 5.13 | 67.53 | 5.64 | 67.50 | 6.26 | 0.351 | |  |
| **Body mass index, kg/m2** | 26.83 | 3.53 | 27.32 | 4.58 | 25.97 | 3.87 | 26.33 | 4.87 | 29.67 | 3.34 | 27.50 | 4.23 | 0.165 | |  |

**Supplementary material Table 3- Overview of demographic characteristics of the study population according to headache type**

1. **Among patients between 50-60 years (n=398)**

|  | | **Categories of headache type** | | | | | | | | | | | | | |
| --- | --- | --- | --- | --- | --- | --- | --- | --- | --- | --- | --- | --- | --- | --- | --- |
|  |  | **Episodic migraine** | | **Tension type headache** | | **Hemicranias continua or cluster headache** | | **MOH and Chronic migraine** | | **HTN-induced headache** | | **Cervicogenic headache** | | **P-value from Chi-square** |  |
|  |  | Count | Column N % | Count | Column N % | Count | Column N % | Count | Column N % | Count | Column N % | Count | Column N % |  |  |
| **Male Sex** | | 5 | 3.0% | 8 | 11.0% | 5 | 29.4% | 7 | 7.7% | 0 | 0.0% | 2 | 8.0% | 0.001 |  |
| **Education** | **< 10 years** | 41 | 24.3% | 25 | 34.2% | 8 | 47.1% | 23 | 25.3% | 4 | 17.4% | 9 | 36.0% | 0.545 |  |
|  | **10-12 years** | 52 | 30.8% | 21 | 28.8% | 3 | 17.6% | 26 | 28.6% | 9 | 39.1% | 7 | 28.0% |  |  |
|  | **12-14 years** | 15 | 8.9% | 6 | 8.2% | 2 | 11.8% | 6 | 6.6% | 4 | 17.4% | 5 | 20.0% |  |  |
|  | **14-16 years** | 40 | 23.7% | 13 | 17.8% | 2 | 11.8% | 22 | 24.2% | 4 | 17.4% | 3 | 12.0% |  |  |
|  | **>16 years** | 21 | 12.4% | 8 | 11.0% | 2 | 11.8% | 14 | 15.4% | 2 | 8.7% | 1 | 4.0% |  |  |
| **Job** | **Employee** | 41 | 24.3% | 14 | 19.2% | 7 | 41.2% | 23 | 25.3% | 8 | 34.8% | 4 | 16.0% | 0.164 |  |
|  | **Self-employed** | 14 | 8.3% | 16 | 21.9% | 4 | 23.5% | 11 | 12.1% | 3 | 13.0% | 5 | 20.0% |  |  |
|  | **retired** | 24 | 14.2% | 12 | 16.4% | 2 | 11.8% | 15 | 16.5% | 5 | 21.7% | 4 | 16.0% |  |  |
|  | **Un-employed/ housewife** | 90 | 53.3% | 31 | 42.5% | 4 | 23.5% | 42 | 46.2% | 7 | 30.4% | 12 | 48.0% |  |  |
| **Marital status** | **Single** | 3 | 1.8% | 4 | 5.5% | 1 | 5.9% | 2 | 2.2% | 0 | 0.0% | 4 | 16.0% | 0.136 |  |
|  | **Married** | 151 | 89.3% | 60 | 82.2% | 16 | 94.1% | 84 | 92.3% | 21 | 91.3% | 19 | 76.0% |  |  |
|  | **Divorced** | 11 | 6.5% | 7 | 9.6% | 0 | 0.0% | 4 | 4.4% | 2 | 8.7% | 2 | 8.0% |  |  |
|  | **Other** | 4 | 2.4% | 2 | 2.7% | 0 | 0.0% | 1 | 1.1% | 0 | 0.0% | 0 | 0.0% |  |  |
| **Smoking** | | 11 | 6.5% | 9 | 12.3% | 4 | 23.5% | 9 | 9.9% | 1 | 4.3% | 1 | 4.0% | 0.235 |  |
| **Opium** | | 1 | 0.6% | 1 | 1.4% | 2 | 11.8% | 2 | 2.2% | 0 | 0.0% | 1 | 4.0% | 0.029 |  |

1. **Among** **patients older than 60 years (n=172)**

|  | | **Categories of headache type** | | | | | | | | | | | | |  | |
| --- | --- | --- | --- | --- | --- | --- | --- | --- | --- | --- | --- | --- | --- | --- | --- | --- |
|  |  | **Episodic migraine** | | **Tension type headache** | | **Hemicranias continua or cluster headache** | | **MOH and Chronic migraine** | | **HTN-induced headache** | | **Cervicogenic headache** | | | **P-value from Chi-square** | |
|  |  | Count | Column N % | Count | Column N % | Count | Column N % | Count | Column N % | Count | Column N % | Count | Column N % |  | |  |
| **Male Sex** | | 3 | 10.3% | 10 | 21.3% | 8 | 36.4% | 5 | 15.2% | 5 | 26.3% | 1 | 4.5% | 0.076 | |  |
| **Education** | **< 10 years** | 9 | 31.0% | 26 | 55.3% | 10 | 45.5% | 15 | 45.5% | 9 | 47.4% | 12 | 54.5% | 0.172 | |  |
|  | **10-12 years** | 4 | 13.8% | 14 | 29.8% | 7 | 31.8% | 8 | 24.2% | 2 | 10.5% | 4 | 18.2% |  |  |  |
|  | **12-14 years** | 4 | 13.8% | 3 | 6.4% | 2 | 9.1% | 4 | 12.1% | 4 | 21.1% | 4 | 18.2% |  |  |  |
|  | **14-16 years** | 7 | 24.1% | 3 | 6.4% | 2 | 9.1% | 5 | 15.2% | 3 | 15.8% | 2 | 9.1% |  |  |  |
|  | **>16 years** | 5 | 17.2% | 1 | 2.1% | 1 | 4.5% | 1 | 3.0% | 1 | 5.3% | 0 | 0.0% |  |  |  |
| **Job** | **Employee** | 2 | 6.9% | 1 | 2.1% | 2 | 9.1% | 1 | 3.0% | 2 | 10.5% | 2 | 9.1% | <0.001 | |  |
|  | **Self-employed** | 1 | 3.4% | 10 | 21.3% | 3 | 13.6% | 0 | 0.0% | 7 | 36.8% | 0 | 0.0% |  |  |  |
|  | **retired** | 16 | 55.2% | 18 | 38.3% | 9 | 40.9% | 14 | 42.4% | 7 | 36.8% | 3 | 13.6% |  |  |  |
|  | **Un-employed/ housewife** | 10 | 34.5% | 18 | 38.3% | 8 | 36.4% | 18 | 54.5% | 3 | 15.8% | 17 | 77.3% |  |  |  |
| **Marital status** | **Single** | 2 | 6.9% | 1 | 2.1% | 0 | 0.0% | 0 | 0.0% | 0 | 0.0% | 0 | 0.0% | 0.755 | |  |
|  | **Married** | 24 | 82.8% | 38 | 80.9% | 20 | 90.9% | 29 | 87.9% | 18 | 94.7% | 18 | 81.8% |  |  |  |
|  | **Divorced** | 1 | 3.4% | 4 | 8.5% | 0 | 0.0% | 2 | 6.1% | 1 | 5.3% | 2 | 9.1% |  |  |  |
|  | **Other** | 2 | 6.9% | 4 | 8.5% | 2 | 9.1% | 2 | 6.1% | 0 | 0.0% | 2 | 9.1% |  |  |  |
| **Smoking** | | 4 | 13.8% | 10 | 21.3% | 3 | 13.6% | 6 | 18.2% | 3 | 15.8% | 3 | 13.6% | 0.942 | |  |
| **Opium** | | 1 | 3.4% | 4 | 8.5% | 1 | 4.5% | 3 | 9.1% | 0 | 0.0% | 1 | 4.5% | 0.720 | |  |

**Supplementary material Table 4- Comorbidities of the study population according to headache type.**

1. **Among patients between 50-60 years (n=398)**

|  | | **Categories of headache type** | | | | | | | | | | | | |  |
| --- | --- | --- | --- | --- | --- | --- | --- | --- | --- | --- | --- | --- | --- | --- | --- |
|  |  | **Episodic migraine** | | **Tension type headache** | | **Hemicranias continua or cluster headache** | | **MOH and Chronic migraine** | | **HTN-induced headache** | | **Cervicogenic headache** | | | **P-value from Chi-square** |
|  |  | Count | Column N % | Count | Column N % | Count | Column N % | Count | Column N % | Count | Column N % | Count | Column N % |  | |
| **Cardiovascular disorders** | | 19 | 11.2% | 8 | 11.0% | 1 | 5.9% | 15 | 16.5% | 3 | 13.0% | 7 | 28.0% | 0.202 | |
| **Hypertension** | | 36 | 21.3% | 14 | 19.2% | 2 | 11.8% | 24 | 26.4% | 14 | 60.9% | 2 | 8.0% | <0.001 | |
| **Thyroid disorders** | **Hypothyroidism** | 43 | 25.4% | 21 | 28.8% | 3 | 17.6% | 36 | 39.6% | 3 | 13.0% | 6 | 24.0% | 0.335 | |
|  | **Hyperthyroidism** | 31 | 18.3% | 14 | 19.2% | 2 | 11.8% | 13 | 14.3% | 6 | 26.1% | 0 | 0.0% |  |  |
|  | **Thyroid nodules** | 4 | 2.4% | 1 | 1.4% | 0 | 0.0% | 2 | 2.2% | 0 | 0.0% | 0 | 0.0% |  |  |
| **Renal disorders** | **Renal failure** | 3 | 1.8% | 1 | 1.4% | 0 | 0.0% | 1 | 1.1% | 2 | 8.7% | 0 | 0.0% | 0.716 | |
|  | **Kidney stones** | 19 | 11.2% | 12 | 16.4% | 2 | 11.8% | 14 | 15.4% | 4 | 17.4% | 3 | 12.0% |  |  |
| **Gastrointestinal disorders** | **diarrhea** | 32 | 18.9% | 6 | 8.2% | 0 | 0.0% | 12 | 13.2% | 2 | 8.7% | 2 | 8.0% | 0.083 | |
|  | **History of gastrointestinal bleeding and/or**  **Peptic/duodenal ulcers** | 22 | 13.0% | 12 | 16.4% | 1 | 5.9% | 19 | 20.9% | 3 | 13.0% | 2 | 8.0% | 0.382 | |
|  | **Irritable bowel syndrome (IBS)** | 49 | 29.0% | 18 | 24.7% | 2 | 11.8% | 19 | 20.9% | 6 | 26.1% | 4 | 16.0% | 0.420 | |
| **Cervical spine disease** | **disc disorders** | 46 | 27.2% | 20 | 27.4% | 2 | 11.8% | 26 | 28.6% | 8 | 34.8% | 9 | 36.0% | 0.108 | |
|  | **Degenerative disorders** | 27 | 16.0% | 4 | 5.5% | 0 | 0.0% | 15 | 16.5% | 0 | 0.0% | 1 | 4.0% |  |  |
| **Clenched teeth / Bruxism** | | 28 | 16.6% | 14 | 19.2% | 7 | 41.2% | 18 | 19.8% | 9 | 39.1% | 1 | 4.0% | 0.008 | |

1. **Among patients older than 60 years (n=172)**

|  | | **Categories of headache type** | | | | | | | | | | | | |  |
| --- | --- | --- | --- | --- | --- | --- | --- | --- | --- | --- | --- | --- | --- | --- | --- |
|  |  | **Episodic migraine** | | **Tension type headache** | | **Hemicranias continua or cluster headache** | | **MOH and Chronic migraine** | | **HTN-induced headache** | | **Cervicogenic headache** | | | **P-value from Chi-square** |
|  |  | Count | Column N % | Count | Column N % | Count | Column N % | Count | Column N % | Count | Column N % | Count | Column N % |  | |
| **Cardiovascular disorders** | | 7 | 24.1% | 17 | 36.2% | 8 | 36.4% | 9 | 27.3% | 8 | 42.1% | 6 | 27.3% | 0.723 | |
| **Hypertension** | | 5 | 17.2% | 22 | 46.8% | 12 | 54.5% | 16 | 48.5% | 17 | 89.5% | 8 | 36.4% | 0.001 | |
| **Thyroid disorders** | **H****ypothyroidism** | 7 | 24.1% | 13 | 27.7% | 4 | 18.2% | 7 | 21.2% | 5 | 26.3% | 4 | 18.2% | 0.155 | |
|  | **Hyperthyroidism** | 7 | 24.1% | 3 | 6.4% | 3 | 13.6% | 11 | 33.3% | 1 | 5.3% | 4 | 18.2% |  |  |
|  | **Thyroid nodules** | 0 | 0.0% | 0 | 0.0% | 0 | 0.0% | 0 | 0.0% | 1 | 5.3% | 1 | 4.5% |  |  |
| **Renal disorders** | **Renal failure** | 0 | 0.0% | 0 | 0.0% | 0 | 0.0% | 0 | 0.0% | 1 | 5.3% | 0 | 0.0% | 0.175 | |
|  | **Kidney stones** | 1 | 3.4% | 4 | 8.5% | 2 | 9.1% | 6 | 18.2% | 1 | 5.3% | 1 | 4.5% |  |  |
| **Gastrointestinal disorders** | **diarrhea** | 2 | 6.9% | 8 | 17.0% | 10 | 45.5% | 3 | 9.1% | 0 | 0.0% | 4 | 19.0% | 0.001 | |
|  | **History of** **gastrointestinal bleeding and/or**  **Peptic/duodenal ulcers** | 1 | 3.4% | 5 | 10.6% | 1 | 4.5% | 6 | 18.2% | 2 | 10.5% | 0 | 0.0% | 0.180 | |
|  | **Irritable bowel syndrome (IBS)** | 8 | 27.6% | 10 | 21.3% | 3 | 13.6% | 10 | 30.3% | 1 | 5.3% | 4 | 18.2% | 0.291 | |
| **Cervical spine disease** | **disc disorders** | 5 | 17.2% | 15 | 31.9% | 8 | 36.4% | 14 | 42.4% | 7 | 36.8% | 6 | 27.3% | 0.596 | |
|  | **Degenerative disorders** | 4 | 13.8% | 5 | 10.6% | 1 | 4.5% | 3 | 9.1% | 3 | 15.8% | 1 | 4.5% |  |  |
| **Clenched teeth / Bruxism** | | 6 | 20.7% | 7 | 14.9% | 5 | 22.7% | 6 | 18.2% | 4 | 21.1% | 5 | 22.7% | 0.631 | |

**Supplementary material Table 5- Categories of depression based on Beck Depression Inventory (BDI) score according to headache type.**

1. **Among patients between 50-60 years (n=398)**

|  | **Categories of headache type** | | | | | | | | | | | | |  |
| --- | --- | --- | --- | --- | --- | --- | --- | --- | --- | --- | --- | --- | --- | --- |
|  | **Episodic migraine** | | **Tension type headache** | | **Hemicranias continua or cluster headache** | | **MOH and Chronic migraine** | | **HTN-induced headache** | | **Cervicogenic headache** | | | **P-value from Chi-square** |
|  | Count | Column N % | Count | Column N % | Count | Column N % | Count | Column N % | Count | Column N % | Count | Column N % |  | |
| **Normal** | 5 | 3.0% | 3 | 4.3% | 1 | 6.3% | 4 | 4.5% | 2 | 8.7% | 2 | 8.3% | 0.262 | |
| **Mild mood disturbance** | 9 | 5.4% | 6 | 8.6% | 1 | 6.3% | 2 | 2.2% | 1 | 4.3% | 0 | 0.0% |  |  |
| **Borderline clinical depression** | 15 | 9.0% | 8 | 11.4% | 0 | 0.0% | 10 | 11.2% | 2 | 8.7% | 0 | 0.0% |  |  |
| **Moderate depression** | 71 | 42.8% | 25 | 35.7% | 7 | 43.8% | 24 | 27.0% | 9 | 39.1% | 9 | 37.5% |  |  |
| **Severe depression** | 50 | 30.1% | 20 | 28.6% | 5 | 31.3% | 32 | 36.0% | 5 | 21.7% | 13 | 54.2% |  |  |
| **Extreme depression** | 16 | 9.6% | 8 | 11.4% | 2 | 12.5% | 17 | 19.1% | 4 | 17.4% | 0 | 0.0% |  |  |

1. **Among patients older than 60 years (n=172)**

|  | **Categories of headache type** | | | | | | | | | | | |  |
| --- | --- | --- | --- | --- | --- | --- | --- | --- | --- | --- | --- | --- | --- |
|  | **Episodic migraine** | | **Tension type headache** | | **Hemicranias continua or cluster headache** | | **MOH and Chronic migraine** | | **HTN-induced headache** | | **Cervicogenic headache** | | **P-value from Chi-square** |
|  | Count | Column N % | Count | Column N % | Count | Column N % | Count | Column N % | Count | Column N % | Count | Column N % |  |
| **Normal** | 2 | 6.9% | 2 | 5.3% | 0 | 0.0% | 3 | 9.4% | 0 | 0.0% | 5 | 23.8% | 0.320 |
| **Mild mood disturbance** | 5 | 17.2% | 1 | 2.6% | 2 | 9.5% | 4 | 12.5% | 2 | 10.5% | 3 | 14.3% |  |
| **Borderline clinical depression** | 4 | 13.8% | 3 | 7.9% | 2 | 9.5% | 3 | 9.4% | 1 | 5.3% | 0 | 0.0% |  |
| **Moderate depression** | 7 | 24.1% | 12 | 31.6% | 6 | 28.6% | 13 | 40.6% | 8 | 42.1% | 4 | 19.0% |  |
| **Severe depression** | 9 | 31.0% | 14 | 36.8% | 9 | 42.9% | 4 | 12.5% | 6 | 31.6% | 7 | 33.3% |  |
| **Extreme depression** | 2 | 6.9% | 6 | 15.8% | 2 | 9.5% | 5 | 15.6% | 2 | 10.5% | 2 | 9.5% |  |

**Supplementary material Table 6- Distribution of patients according to categories of sleep apnea total score based on STOP-BANG questionnaire.**

1. **Among patients between 50-60 years (n=398)**

|  | **Categories of headache type** | | | | | | | | | | | |  | |
| --- | --- | --- | --- | --- | --- | --- | --- | --- | --- | --- | --- | --- | --- | --- |
|  | **Episodic migraine** | | **Tension type headache** | | **Hemicranias continua or cluster headache** | | **MOH and Chronic migraine** | | **HTN-induced headache** | | **Cervicogenic headache** | | **P-value from Chi-square** | |
|  | Count | Column N % | Count | Column N % | Count | Column N % | Count | Column N % | Count | Column N % | Count | Column N % |  |  |
| **Low risk of OSA** | 161 | 95.3% | 57 | 78.1% | 15 | 88.2% | 70 | 76.9% | 16 | 69.6% | 25 | 100.0% | <0.001 |  |
| **Intermediate risk of OSA** | 8 | 4.7% | 16 | 21.9% | 2 | 11.8% | 21 | 23.1% | 7 | 30.4% | 0 | 0.0% |  |  |

1. **Among patients older than 60 years (n=172)**

|  | | **Categories of headache type** | | | | | | | | | | | | | | | | | | | | | | | |  |
| --- | --- | --- | --- | --- | --- | --- | --- | --- | --- | --- | --- | --- | --- | --- | --- | --- | --- | --- | --- | --- | --- | --- | --- | --- | --- | --- |
|  |  | **Episodic migraine** | | | | **Tension type headache** | | | | **Hemicranias continua or cluster headache** | | | | **MOH and Chronic migraine** | | | | **HTN-induced headache** | | | | **Cervicogenic headache** | | | | **P-value from Chi-square** |
|  |  | Count | | Column N % | | Count | | Column N % | | Count | | Column N % | | Count | | Column N % | | Count | | Column N % | | Count | | Column N % | |  |
| **Low risk of OSA** | 28 | | 96.6% | | 36 | | 76.6% | | 15 | | 68.2% | | 26 | | 81.3% | | 7 | | 36.8% | | 18 | | 81.8% | | <0.001 | |
| **Intermediate risk of OSA** | 1 | | 3.4% | | 11 | | 23.4% | | 7 | | 31.8% | | 6 | | 18.8% | | 12 | | 63.2% | | 4 | | 18.2% | |  | |
